# Autophagy as a Mechanism for Adaptive Prediction-Mediated Emergence of Drug Resistance

**DOI:** 10.3389/fmicb.2021.712631

**Published:** 2021-09-10

**Authors:** Nivedita Nivedita, John D. Aitchison, Nitin S. Baliga

**Affiliations:** ^1^Institute for Systems Biology, Seattle, WA, United States; ^2^Seattle Children’s Research Institute, Seattle, WA, United States

**Keywords:** adaptive prediction, conditional fitness, drug resistance, autophagy, structured environments

## Abstract

Drug resistance is a major problem in treatment of microbial infections and cancers. There is growing evidence that a transient drug tolerant state may precede and potentiate the emergence of drug resistance. Therefore, understanding the mechanisms leading to tolerance is critical for combating drug resistance and for the development of effective therapeutic strategy. Through laboratory evolution of yeast, we recently demonstrated that adaptive prediction (AP), a strategy employed by organisms to anticipate and prepare for a future stressful environment, can emerge within 100 generations by linking the response triggered by a neutral cue (caffeine) to a mechanism of protection against a lethal agent (5-fluoroorotic acid, 5-FOA). Here, we demonstrate that mutations selected across multiple laboratory-evolved lines had linked the neutral cue response to core genes of autophagy. Across these evolved lines, conditional activation of autophagy through AP conferred tolerance, and potentiated subsequent selection of mutations in genes specific to overcoming the toxicity of 5-FOA. These results offer a new perspective on how extensive genome-wide genetic interactions of autophagy could have facilitated the emergence of AP over short evolutionary timescales to potentiate selection of 5-FOA resistance-conferring mutations.

## Introduction

Antimicrobial resistance has become a major threat to modern medicine with ever adapting strains of microbes and evolution of sub-populations of treatment resistant cells. According to CDC, in the U.S. alone, at least 2.8 million people per year contract an antimicrobial-resistant infection (Magill et al., 2014). Drug resistance is also a growing concern in the treatment of cancers. For the development of efficacious treatment regimen, it has, therefore, become imperative to determine the evolutionary trajectories and associated mechanisms that enable the selection of rare resistance-conferring mutations. Recent evidence has shown that selection of resistance conferring mutations is potentiated by a preceding tolerant state that may manifest from drug-induced physiological adaptation or mutations in generalized tolerance networks (Peterson et al., 2016; Levin-Reisman et al., 2019). Here we have investigated how emergence of resistance may be potentiated by adaptive prediction (AP), a phenomenon utilized by all organisms to anticipate and prepare in advance for a future environmental change (Brunke and Hube, 2014; Mitchell and Lim, 2016; de Lomana et al., 2017). For example, *E. coli* and *M. tuberculosis* sense neutral cues such as rise in temperature or a drop in oxygen to adaptively predict a hostile host environment (Tagkopoulos et al., 2008; Brunke and Hube, 2014; Mitchell and Lim, 2016). In other words, AP may allow an organism to transiently tolerate stressful environments including the lethal effects of drugs, thereby increasing the likelihood of selecting drug-specific resistance mutations.

**FIGURE 1:**
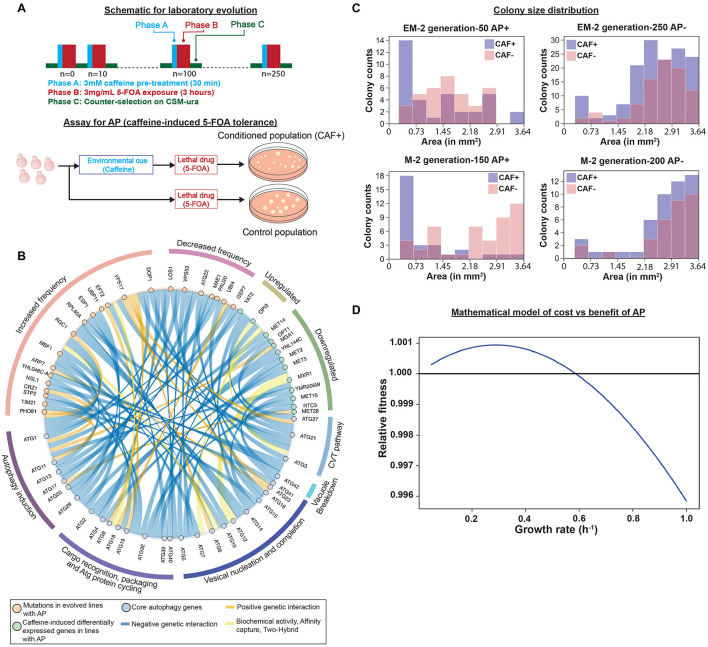
Regulation of emergence of adaptive prediction (AP) during laboratory evolution. **(A)** Experiment design for laboratory evolution of caffeine-induced AP of increased conditional fitness to 5-FOA. (adapted from our previous work de Lomana et al., 2017). **(B)** Genes with increased or decreased mutation frequency or perturbed regulation in evolved lines with AP interact genetically, physically, or biochemically with core autophagy genes (re-analysis of data previously published in de Lomana et al., 2017). **(C)** Distribution of colony sizes in EM2 (engineered line that was UV-mutagenized) and M2 (parental strain- BY4741-URA3 that was UV-mutagenized) evolved lines. EM2 line exhibited AP from generation 50 to 200 and AP was lost at generation 250. M2 line gained AP by generation 100, retained AP through generation 150, and ultimately lost it by generation 200. Colony size distribution indicates presence of smaller sized colonies in caffeine-treated (CAF+) cultures of evolved lines with AP (AP+, EM2: generation 50; and M2: generation 150) or without AP (AP−; EM2: generation 250; and M2: generation 200), as indicated in the header of each plot. Colony sizes were measured using Image J (Weka segmentation) and pixel area was converted to mm^2^ based on total pixel area of the entire plate. **(D)** Relationship between growth rate and relative fitness inferred by a mathematical model of cost vs. benefit of AP.

Previously, we demonstrated that repeated exposure to a neutral cue followed by a sub-lethal dose of a toxin drives the emergence of AP-mediated tolerance over short evolutionary cycles (de Lomana et al., 2017). Specifically, over every 10 generations we subjected *S. cerevisiae* (yeast) to pre-conditioning with 3 mM caffeine (a neutral cue that had no effect on growth) followed by a sub-lethal dose of 3 mg/mL 5-fluoroorotic acid (5-FOA), which kills ∼80% of cells in the population (Figure 1A; de Lomana et al., 2017). 5-FOA is toxic to yeast because upon conversion by Ura3 to 5-fluorouracil (5-FU, a pyrimidine analog), 5-FU is misincorporated into RNA and DNA in place of uracil. The coupled treatments with caffeine and 5-FOA were interspersed with a period of counter-selection in a growth medium without uracil to weed out 5-FU resistant uracil auxotrophs. The cyclic exposure to caffeine followed by 5-FOA, resulted in emergence of AP within 50–150 generations across all evolved lines (de Lomana et al., 2017). Remarkably, across nearly all evolved lines, AP was eventually displaced by a population that had higher overall resistance to 5-FOA. It was also investigated whether AP could emerge from rewiring of regulatory and signaling networks. Specifically, URA3 was translationally fused to a peroxisome targeting signal (from GPD1-Glycerol-3-Phosphate Dehydrogenase), giving the engineered yeast strain (referred to as EM-strain in this work) the potential to acquire AP by rewiring the caffeine response to a signaling network that regulates protein translocation to the peroxisome. Remarkably, within 50–150 generations few evolved lines utilized caffeine-induced translocation of URA3 to peroxisomes to gain conditional 5-FOA tolerance, demonstrating that AP can emerge from rewiring of regulatory and signaling networks. In the engineered line, EM4, fluorescence microscopy revealed caffeine-dependent re-localization of GPD1-EGFP-Ura3 to peroxisomes, which would confer resistance to 5-FOA (phase b) without compromising the ability to grow in the absence of uracil, due to a cytosolic pool, in the counter-selection step (phase C) of the laboratory evolution cycle (Figure 1A). This translocation disappeared by generation 250 when AP was lost in the strain. However, not all engineered lines evolved AP through this rewiring mechanism suggesting the existence of additional mechanisms for AP-mediated 5-FOA tolerance (de Lomana et al., 2017). In addition to EM lines, AP was also observed during laboratory evolution of UV-mutagenized cultures of the parental BY4741-URA3 strain (referred to as M-lines in this work) as well as clonal populations of the parental BY4741-URA3 strain, suggesting that yeast was capable of gaining AP at high frequency through some generalized mechanism(s). In this work, we analyzed selected mutations and differentially expressed genes in evolved lines and discovered that the mechanism of AP across multiple evolved lines could be explained by rewiring of the caffeine response to the autophagy network.

## Results and Discussion

To determine mechanisms of AP, we analyzed whole genome sequences and RNA-seq data for up to 5 evolved lines across generations over which they gained AP (Supplementary Data from de Lomana et al., 2017). This analysis discovered mutations in 95 genes (90 genes with ≥ 20% frequency) and caffeine-induced > 2-fold differential expression of 39 genes that may have contributed to the AP phenotype. Although caffeine is known to target DNA replication and repair (Tsabar et al., 2015), none of the genes of this pathway were mutated across any evolved line. However, using YeastMine (Balakrishnan et al., 2012) we discovered that 13 differentially expressed genes either regulate or genetically interact with AuTophaGy (ATG) genes (Figure 1B). Notably, 9 out of 11 downregulated genes have negative genetic interactions with ATG genes, suggesting that caffeine-induced downregulation of these genes might have induced autophagy. In parallel, using PROVEAN (Choi and Chan, 2015) to infer consequences of mutations on protein function, we discovered 24 deleterious/non-synonymous and upstream intergenic mutations in 34 genes (including non-synonymous mutation in VPS17 and upstream gene variant in ATG22- mutated in multiple lines) that genetically interact with ATG genes (Figure 1B). We performed hypergeometric tests to determine significance of interactions of 34 mutated genes and 13 differentially expressed genes with autophagy, which is a hub in the genetic interaction network of yeast. Specifically, the 33 genes of the core autophagy process genetically interact with 1,886 out of 6,604 genes in the yeast genome. Hence, the hypergeometric test yielded *p*-values of 0.9 for mutated genes and 0.4 for differentially expressed genes. Although this did not meet a statistically significant level of enrichment, the large number of genes associated with autophagy reflects the fact that autophagy is associated with many cellular processes and suggests a high likelihood for many mutations selected during evolution to influence autophagy, if it confers a fitness benefit. This may reflect the central role of autophagy in AP and many other cellular processes. Therefore, we performed phenotypic analysis to investigate whether rewiring of the caffeine-response to induction of autophagy could have potentially contributed to AP in some of the evolved lines.

Autophagy is an evolutionarily conserved process for recycling amino acids and nutrients from damaged proteins and other cellular macromolecules to support cell survival in stressful environments (Neufeld, 2012; Rangarajan et al., 2020; Pang et al., 2021). In yeast, induction of autophagy is associated with decreased growth rate, which manifests in smaller sized colonies in stressful or lethal environments (Neufeld, 2012). Strikingly, we observed that caffeine pre-treatment resulted in higher numbers of smaller colonies, in generations when the evolved line exhibited AP (Figure 1C). Further, we used a deterministic model based on work by Mitchell and Pilpel (2011) and adapted it for yeast growth characteristics. The model focuses on two response strategies- “conditional response” (i.e., response to 5-FOA after caffeine pre-treatment) and “direct response” (i.e., the response to 5-FOA without caffeine pre-treatment). “Benefit” and “cost” associated with the two response strategies were defined as a function of the associated change in basal growth rate. “Relative fitness” due to caffeine pre-treatment was calculated by subtracting direct response from conditional response (Mitchell and Pilpel, 2011). Using this model, we determined that reduction in growth rate in response to a preceding neutral cue could confer higher conditional fitness to a future stressful environment, bolstering caffeine-induced autophagy as a possible mechanism for AP (for details please refer to the Supplementary File) (Figure 1D).

**FIGURE 2:**
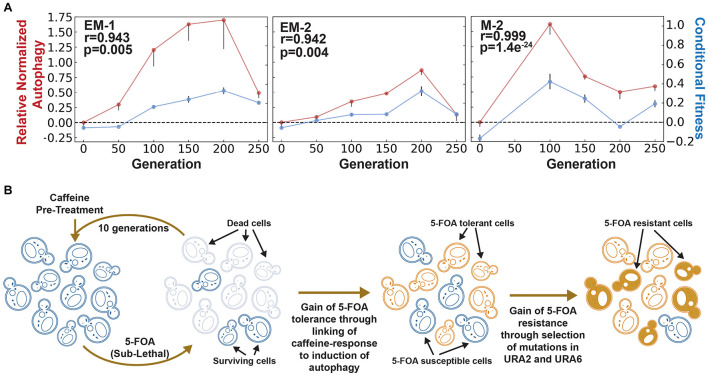
Evaluation of autophagy as a mechanism for adaptive prediction. **(A)** Relative normalized autophagy and conditional fitness in EM and M cell lines. AP emerged within 100–150 generations in all evolved lines. Spearman correlation (r) between conditional fitness and relative normalized autophagy, and associated significance (*p*-value), is indicated for each evolved line. **(B)** Model for how rewiring of a neutral cue-induced response to autophagy is a generalized mechanism for generating AP-based tolerance and subsequent resistance to a lethal stress. Rise of a tolerant population due to AP could ultimately potentiate development of resistance through subsequent selection of mutations in specific genes and pathways targeted by the lethal stress (in this example, mutations in Ura2/Ura6 genes of uracil biosynthesis pathway for constitutive resistance to 5-FOA).

To generate definitive evidence that AP had emerged from rewiring of caffeine response to the autophagy network, we performed the Rosella assay using a dual-color emission biosensor (Rosado et al., 2008). The Rosella biosensor consists of two tandem fluorescent proteins- a pH sensitive green fluorescent protein (GFP) and a pH stable red fluorescent protein (RFP). This biosensor is targeted to the pH neutral cytosol under normal conditions, wherein both GFP and RFP are active. Upon induction of autophagy, the biosensor is translocated to an acidic vacuole wherein the pH-sensitive GFP is selectively inactivated and only the RFP remains active. Thus, the ratio of red to green fluorescence can be used as a measure of bulk autophagy (Rosado et al., 2008; Rangarajan et al., 2020). Using this assay, we determined that autophagy was induced upon caffeine pre-treatment across multiple evolved lines in generations exhibiting AP, but not in parental cultures (i.e., 0th generation) or in generations when AP was lost. In fact, the longitudinal patterns of changes in caffeine-induced autophagy and conditional fitness to 5-FOA were remarkably correlated (Figure 2A). Together with the demonstration that 3 mM caffeine had no effect on growth of yeast (de Lomana et al., 2017), these results rule out the possibility that caffeine pre-treatment might have directly triggered autophagy by modulating the TORC pathway (Reinke et al., 2006; Winter et al., 2008). Thus, our results demonstrate that AP had emerged through the selection of mutations that linked the caffeine-response to autophagy to adaptively predict and increase survivability to 5-FOA. Notably, the loss of AP across the evolved lines was accompanied by correlated reduction in caffeine-induced autophagy and an overall increase in resistance to 5-FOA through the selection of mutations in similar sets of genes, including URA2 and URA6 (Supplementary Figure 2 and Supplementary Table 1), which likely reduced flux through uracil biosynthesis pathway (Flynn and Reece, 1999).

While the caffeine induction of autophagy may have reduced flux through uracil biosynthesis pathway by generating uridine phosphates (Guo et al., 2016), evidence generated in this study has demonstrated that autophagy-mediated reduction in growth rate is also a plausible mechanism of 5-FOA tolerance. As noted previously, 1,886 of the 6,604 total genes in the yeast genome genetically interact with the 33 genes that are implicated in the core autophagy process, explaining why multiple evolved lines had converged on the same solution for AP-mediated 5-FOA tolerance. Hence, transient gain of AP through conditional induction of autophagy appears to be a generalized mechanism that enabled yeast across multiple evolved lines to gain 5-FOA resistance by selecting rare mutations in similar genes, without compromising its ability to grow in the absence of uracil during the counter-selection step (phase C –see Figure 1A; de Lomana et al., 2017).

We propose that pathogens might exploit rewiring of host-relevant environmental cues to autophagy as a mechanism to rapidly evolve AP-mediated generalized tolerance to diverse lethal agents. The advanced preparedness through conditional induction of autophagy creates a window of opportunity for cells to encounter and select resistance-conferring mutations in genes directly associated with the mechanism of action of a given drug (Figure 2B). This model offers an adaptive evolutionary perspective for the wide association of autophagy with the development of drug resistance (Levine and Kroemer, 2008), and extends implications of this phenomenon to emergence of resistance in pathogens. Further, the model also proposes that autophagy suppressor drugs could improve efficacy of treatment regimen by blocking a major evolutionary pathway for gain of drug resistance (Yang et al., 2011).

## Materials and Methods

### Yeast Strains, Culturing, and Conditional Fitness Measurements

Strains used in this study were derived from BY4741-URA3 strain of *S. cerevisiae*, as reported previously (de Lomana et al., 2017). “EM” refers to BY4741 GPD1-EGFP-URA3 strain that has undergone mutagenesis with UV light (Stratalinker UV Crosslinker Model 2,400 at 9,300 mJ cm^–2^ with 20% cell survival). UV mutagenesis was used to introduce genetic variation to generate mixed population of genotypes. BY4741 GPD1-EGFP-URA3 strain was constructed by genomic integration of a PCR fragment containing EGFP and URA3 in-frame, amplified using the plasmids-pYM27 and pRS426. “M” refers to UV-mutagenized culture of the parental BY4741-URA3 strains mutagenized as described earlier. Glycerol stocks for generations 0–250 for each evolved line were revived on agar plates, cultured to log-phase in complete synthetic media without uracil with 2% glucose, at 30°C on a shaker. Cell density was determined by hemocytometer counts. Conditional fitness assays were performed as described by de Lomana et al. (2017). In brief, culture aliquots were subjected to a sub-lethal dose of 5-FOA (3 mg/mL) for 3 h, with and without 30 min of pre-conditioning with 3 mM caffeine. Conditional fitness (CF) is calculated as difference between survival with caffeine pretreatment (Sc) and survival without caffeine pre-treatment or control (*S*_*o*_). S (survival) is calculated as:


(1) $$S={\bar{x}}_{post-FOA}/{\bar{x}}_{pre-FOA}$$

where ${\bar{x}}_{pre-FOA}and{\bar{x}}_{post-FOA}$ are average CFU (Colony formation unit) counts before and after 5-FOA treatment.

### Autophagy Measurement

Cells from generations 0 to 250 for each evolved line were transformed with Rosella biosensor (pAS1NB c Rosella I—Addgene plasmid #71245) using the yeast transformation kit (Sigma Aldrich). The cells were cultured to log phase and then subjected to sub-lethal dose of 5-FOA (3 mg/mL) for 3 h, with and without (Control) 30 min of pre-conditioning with 3 mM caffeine. After 3 h of 5-FOA treatment, the samples (both control and caffeine-pre-conditioned) were concurrently evaluated for AP and autophagy. For autophagy measurement, 200 μL of cells from each sample were aliquoted into a Nunc flat bottomed 96-well plate. Red and green fluorescence of each well was measured using a plate reader (Synergy H4 Hybrid Reader). Excitation and emission settings for quantifying red fluorescence (DsRed) were set to 543 nM (excitation) and 587 nm (emission). For green fluorescence (Super ecliptic pHluorin), 488 nm was set for excitation and 530 nm for emission. After subtraction of background fluorescence, overall autophagy was calculated as the difference in ratio of average red to green fluorescence, with and without (control) caffeine pre-treatment, and plotted in reference to overall autophagy in the parental population. Autophagy difference (A_*d*_) was calculated as follows:


(2) $$A_{d}=A_{normalized-Caffeine}-A_{normalized-Control}$$

where *A*_*normalized–Control*_ and *A*_*normalized–Caffeine*_ are autophagy measurements normalized to the average autophagy in the parental population. Relative normalized autophagy was then determined relative to A_*d*_ at Generation 0.

## Data Availability Statement

The data underlying this article is available in the article, the Supplementary Data file and the supplementary data of the previously published work by de Lomana et al. (2017).

## Author Contributions

NN, JA, and NB designed the study. NN performed the experiments and data analysis. NN and NB drafted the manuscript. All authors wrote the manuscript.

## Conflict of Interest

The authors declare that the research was conducted in the absence of any commercial or financial relationships that could be construed as a potential conflict of interest.

## Publisher’s Note

All claims expressed in this article are solely those of the authors and do not necessarily represent those of their affiliated organizations, or those of the publisher, the editors and the reviewers. Any product that may be evaluated in this article, or claim that may be made by its manufacturer, is not guaranteed or endorsed by the publisher.
