# Supplementary material for: Autophagy as a Mechanism for Adaptive Prediction-Mediated Emergence of Drug Resistance
Source: Front Microbiol. 2021 Sep 10;12:712631. doi: 10.3389/fmicb.2021.712631 (PMC8461305; doi:10.3389/fmicb.2021.712631)
Supplement: Supplementary file 1 [file Data_Sheet_1.PDF]

Supplementary information for

Autophagy as a mechanism for adaptive prediction-mediated emergence of drug resistance

Mathematical model

Based on the work by Mitchell, A., & Pilpel, Y. (2011). A mathematical model for adaptive prediction of environmental changes by microorganisms. Proceedings of the National Academy of Sciences of the United States of America, 108(17), 7271–7276. https://doi.org/10.1073/pnas.1019754108

Relative fitness is calculated by determining the cost and benefit associated with both conditional response (CR) (response to 5-FOA after caffeine pre-treatment) and direct response (DR) (response to only 5-FOA). The gain and cost functions are determined as dynamic changes in the basal growth rate.

At steady state:

Change in basal growth rate (δ):

$$\delta = b.c.\delta_{basal}$$

Where δ_{basal} is the basal growth rate, *b* is the benefit from the response and *c* is the cost of mounting the response.

Fitness (*F*) of an organism can then be defined as sum of all changes relative to the basal growth rate:

$$F = \int_0^\infty b(t).c(t)dt$$

Relative response, *r* (normalized to steady state level of protein):

Relative response for Direct response, $r_{DR}(t) = \begin{matrix} 0 & \quad t \leq \Delta t \\ 1 - e^{-\alpha t} & \quad t > \Delta t \end{matrix}$

Relative response for conditional response, $r_{CR}(t) = 1 - e^{-\alpha t}$

Where *t* is the time from induction, *α* is the degradation rate (for the protein level generated as a response) and *Δt* is the duration of caffeine pre-treatment.

Benefit is then defined by (individually for direct response and conditional response):

$$b_{DR}(t) = 1 + \kappa \, r_{DR}(t)$$

$$b_{CR}(t) = \begin{matrix} 1 & \quad t \leq \Delta t \\ 1 + \kappa \, r_{DR}(t) & \quad t > \Delta t \end{matrix}$$

Where κ is the scaling parameter for relative growth advantage (determined by the type of organism). We used the average conditional fitness advantage of 0.275 (determined from our previous work in *de Lomana et al., 2017*).

Cost is defined by:

$$c_{DR}(t) = \begin{cases} 1 & t \leq \Delta t \\ 1-\eta & t > \Delta t \end{cases}$$

$$c_{CR}(t) = 1-\eta$$

Where η is a system scaling factor which in our case is defined as survivability of the population on exposure to 5-FOA (~20%).

From equations of fitness function and benefit and cost function, we can solve the integral of fitness function for lab evolution (coupled response- caffeine followed by 5-FOA) as:

$$\Delta F = \kappa\,(1-\eta)\frac{(1-e^{-\alpha\Delta t})}{\alpha} - \eta\Delta t$$

In Fig.1d, we plot relative fitness or population ratio, which is defined as:

$$RF = \exp\,(\delta_{basal}.\Delta F)$$

RF above 1 indicates fitness advantage in a coupled environment (i.e. pre-treatment followed by sub-lethal dose).

Figure showing reduced autophagy expression in AP+ line (M1):

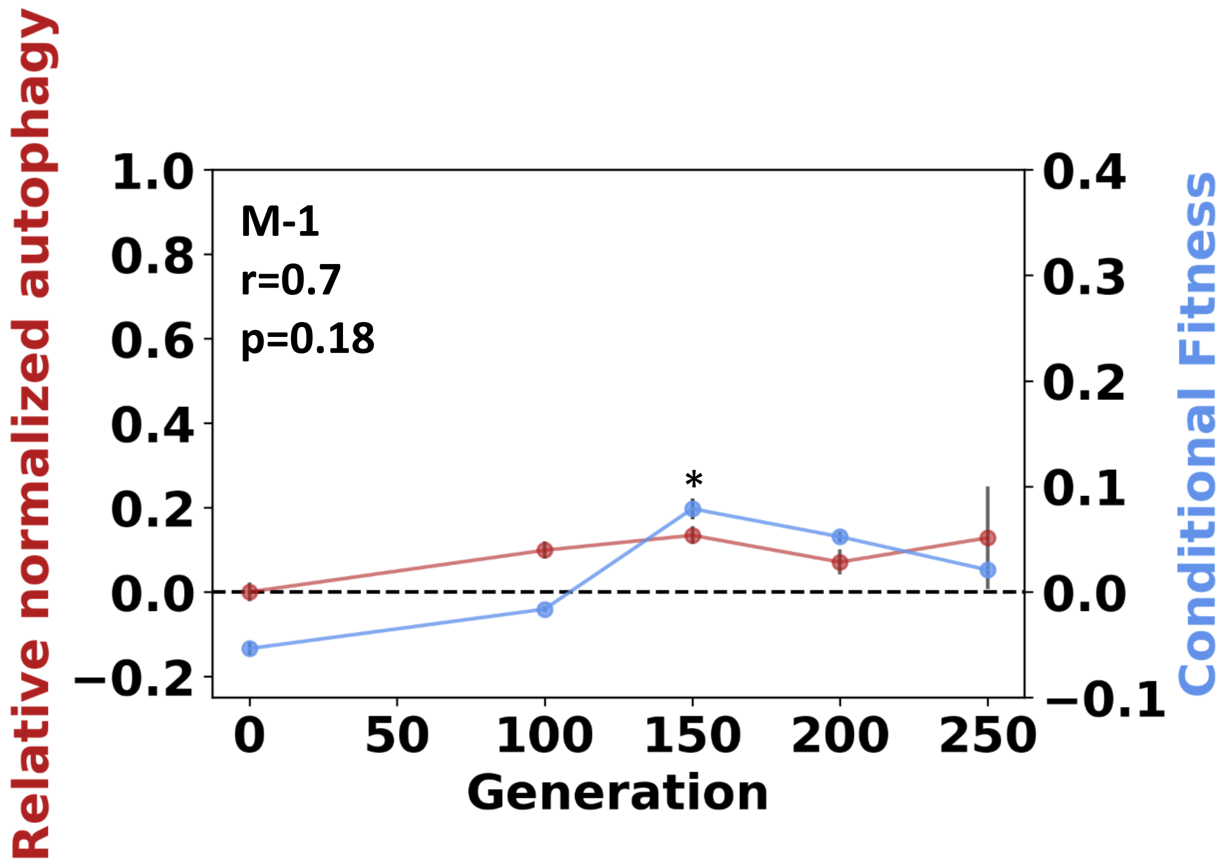

Figure S1 Autophagy expression is not always correlated to the conditional fitness in an evolved line. We evaluated autophagy and conditional fitness for evolved line M1, where the autophagy expression and conditional fitness was not correlated (spearman correlation-pvalue=0.18), indicating that not all lines utilize autophagy as a mechanism for conditional increase in fitness or resistance to 5-FOA.

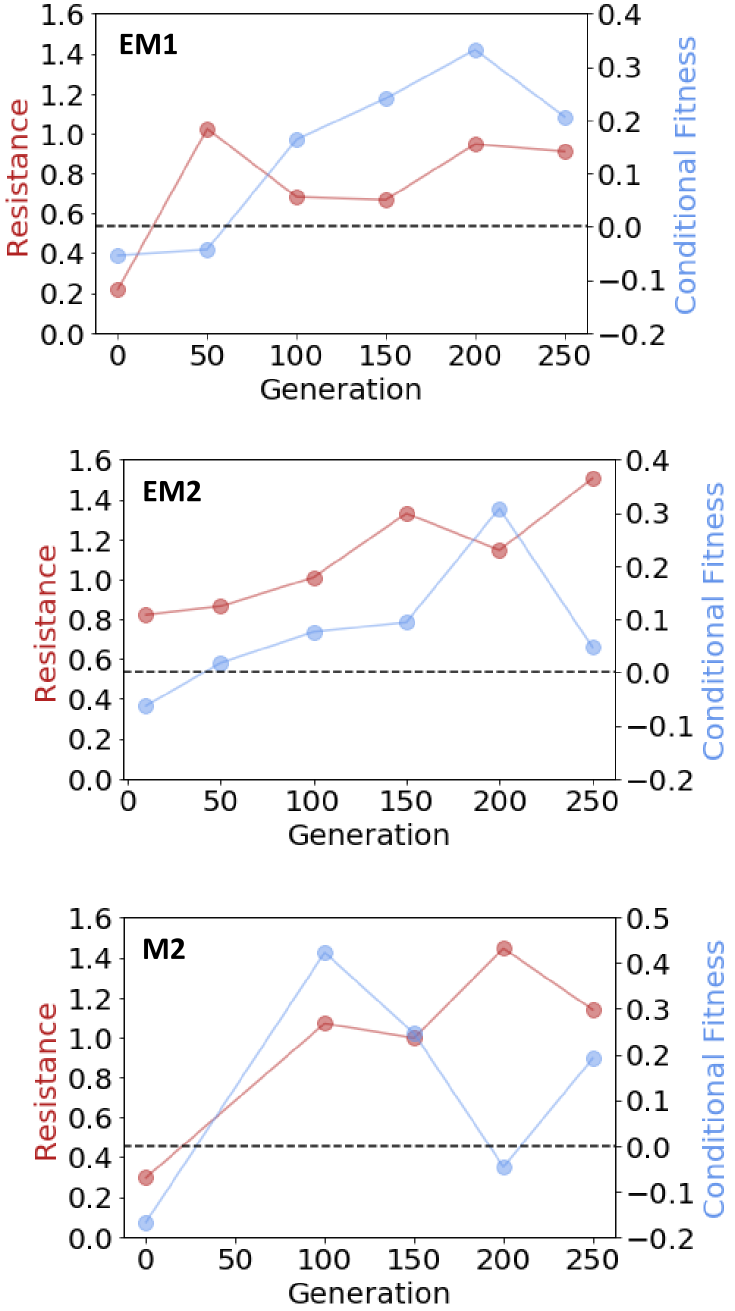

Figure S2 **5-FOA resistance increases over the course of the lab evolution experiment.** We used colony formation unit count (similar to our method of determining conditional fitness) to determine constitutive resistance for the lines EM1, EM2 and M2 over the course of lab evolution experiment. The plots show overall increase in resistance to 5-FOA, even as AP is lost after a few generations. This indicates that rise of constitutive resistance becomes an alternate strategy of the population.

Mutations	Evolved lines
ATG22	EM6, C1
URA2	EM2, EM4, EM6, C1, M1
LOS1	M1, C1
UBP11	EM2, EM4, C1
PHO81	EM6, M1
TIM21	EM2, C1
UBI4	M1, C1
YBT1	M1, C1
ERR2	M1, EM2, EM6

Table S1. Mutations (>20% frequency) in multiple evolved lines with AP. EM2, EM4 and EM6 refers to 6 engineered and UV mutagenized evolved lines. M1 and M2 refer to UV mutagenized evolved lines and C1 refers to wild-type line (not mutagenized).
